# Kazakh “free women” grit—Chinese Kazakh women's clothing image in the context of multicultural integration of silk road

**DOI:** 10.3389/fpsyg.2022.873815

**Published:** 2022-07-26

**Authors:** Rui Xu

**Affiliations:** College of Art, Jinling Institute of Technology, Nanjing, China

**Keywords:** Silk Road, multiculture, Kazakh, women's clothing, clothing image

## Abstract

In recent years, Chinese clothing cultural heritage and knowledge genealogy along the Silk Road have become the research headline attracting public attention. In particular, Kazakh clothing in Northwestern China has become the focus of today's traditional national culture. Kazakh, located at the intersection of the Silk Road, has an important position. The traditional clothing made by various social factors reflects the style and identity integration throughout history in cultures along the Silk Road, taking women's clothing as an example. Kazakh women are good at crafts, which is reflected in their strong grit. They gradually changed their role and traditional values, which has guided their social role. At the same time, it also reflects the awakening of Kazakh women's feminist consciousness to a large extent, and complies with Confucianism, along with the mainstream value of “advanced gender culture” of the world's outstanding civilization achievements. This paper summarizes the form, color, and patterns of Kazakh women's clothing by analyzing the garment, headdress, and footwear in Xinjiang Uygur Autonomous Region Museum, Aksai Kazakh National Museum, and Capital Museum of China. Through the analysis, this paper further probes into the multicultural integration between Kazakh and the regions along the Silk Road and interprets the idea represented by the forms, colors, and patterns in the clothing to promote the inheritance and development of traditional and regional cultures together with ethnic features along the Silk Road.

## Introduction

“Silk Road” clothing art, a mirror of material and spiritual civilizations of the times, reflects the integration of historical culture and modern civilization of all regions and nationalities. Ancient ethnic groups, such as the Kazakhs and their ancestors, lived along the Silk Road as builders and operators of the ancient Silk Road and were also the beneficiaries of the economic and cultural prosperity brought by the Silk Road (Fan, 2007). The origin of the Kazakh ethnic group is relatively complex. They were mainly formed by the fusion of the ancient Wusun, Kangju, Alans, the Sakas who were originally in the Central Asian grasslands, people in Central Asia, and the Huns who entered this area later, Xianbei, Rouran, and Tujue, Tiele, Khitan, Mongolian, and other ethnic groups (Xinjiang Uygur Autonomous Region Association for foreign cultural exchanges, 2012). Both the ancient “Silk Road” and today's “Silk Road Economic Belt” demonstrate the frequent cultural exchanges between the Chinese Kazakh ethnicity and surrounding countries. Clothing with distinct ethnic and historical features has played a distinct role in the interactions between Kazakh and other regions. Though Kazakh is under the influence of customs and spiritual cultures in Northwestern China, it retains its uniqueness while absorbing foreign cultures. Women's clothing which is decorated with various colors and embroidery contains religious symbols signifying prosperity while imparting an aesthetic impression (Duan, 2004). Their accessories like headdresses and boots are also functional for carrying things like knives and perfume pouches, while they protect the body from the cold and/or bear a religious significance to the region (Zhong and Fan, 2006). As a result, Kazakh clothing and dressing style along with Xiyu (the western region of China) style was more than the epitome of the “Oriental Road” civilization as it also mixed the characteristics of apparel from different countries on the Eurasian continent.

However, nowadays, despite frequent cultural exchanges and high-speed information dissemination, there are fewer opportunities to see Kazakh traditional ethnic dress, and even Kazakh women only wear them on specific occasions or when attending festivals. This type of ethnic costume is decorative and reflects their culture in every detail of the costume, such as hat, collar, sleeve, skirt edge, belt, and footwear. The ornaments on the clothing are designed based on practical usage and are represented by the style, color, and pattern, which proved that both clothing and accessories make up an integral clothing system of a nation. Kazakh Chinese generally worship the ancestral mother (Dai, 1994), or Mother Earth till today. Therefore, the matrilineal society vestige has been retained in Kazakh, as recorded incisively and vividly in the *Dayuan Liezhuan of the Historical Records* (Sima, 2006). The recorded history shows that women had absolute discourse power and high status in ancient Dayuan State, contributing to the prominent design in women's clothing. Significantly, the headdress and footwear of Kazakh women play an irreplaceable role in their nomadic life. This paper focuses on Kazakh women's garments, headdresses, and footwear by their form, color, and decorations to interpret the clothing characteristics demonstrated in the integration and absorption of Kazakh grassland culture with the ancient Chinese Zhongyuan, or Central Plain culture, Central Asian culture, and Western Asian culture. Besides, the research also delves into the cultural implications behind Kazakh women's clothing.

The evolution of Chinese Kazakh women's clothing is closely related to the cultural exchange between different regions along the Silk Road. Research on the form, colors, and decorations of their traditional clothing is abundant in domestic and overseas studies relating to clothing and about ethnic minorities. However, the cultural heritage of Kazakh women's clothing in relation to the “silk road” is worth an in-depth study. In particular, their “clothing, headdress, and footwear” need further examination within an academic framework. Previous research on this theme include a study classifying male and female headdresses of Kazakh, which are found throughout China, according to the region, and analyzing them depending on each religion (Kim, 2001). Another study classifies women's headdresses among the Muslim minority in Xinjiang autonomous region by type and explores the reasons for wearing them (Kang and Cho, 2015). A couple of studies selected Kazakh in the northwestern regions of China and analyzed the characteristics of their garments and accessories (Kwon, 1995; Park, 1995; Han and Cho, 1997), and documented changes in Kazakh's traditional clothing (Lu, 2017). Zi (2009) analyzed the use of color by classifying married and unmarried women's clothing. A study by Shang (2020), developed a design utilizing the patterns in Kazakh clothing. However, from a feminist perspective, research on Kazakh women's clothing in the context of the multicultural integration of the Silk Road is insufficient. Our research aims to address that need.

Therefore, the purpose of this study is to understand the background of the formation of clothing cultures for Chinese Kazakh and to provide a theoretical framework to help preserve their cultural heritage as expressed through their women's attire.

As for the research method, the literature review and case study were conducted together. The literature review included books, academic journals, and academic papers related to the clothing of minorities in China, particularly the clothing of Kazakhs. A total of 90 items were collected by selecting 30 each for women's garment, headdress, and footwear types and were used in analyzing the status and social structure of Kazakh women in social life. Cases were primarily collected by visiting Xinjiang Uygur Autonomous Region Museum, Aksai Kazakh National Museum, and Capital Museum. There are several Chinese museums that house ethnic costumes, like the Shanghai Museum, Ethnic Museum, Xinjiang Uygur Autonomous Region Museum, Xinjiang Aksai Kazakh National Museum, and the Capital Museum. But the latter three have the largest number of Kazakh costumes, more than 150 pieces, so they were selected for the case study visits. Secondary data were extracted from books related to domestic and Chinese minorities' clothing history and the Internet. To ensure objectivity, an evaluation group consisting of five scholars with PhDs in clothing and textile majors analyzed and categorized the 90 garments and accessories of Kazakh women, based on the characteristics of the clothing item image.

## The cultural origin of Kazakh women's clothing in multicultural integration

Chinese Kazakh live in the region at the center of the Eurasian continent. To be specific, their habitat is at the junction of Kazakhstan, Tajikistan, Pakistan, Kyrgyzstan, and other central Asian countries, serving as a hub on the Silk Road. The *Book of the Later Han* recorded the history of the Silk Road and how ancient Chinese merchants reached Central Asia and Western Asia using the Silk Road. Upon their arrival and settlement, those merchants introduced exotic cultures from the Central Plains, the Arab-Islam, and other civilizations into the Xinjiang region. Interspersed with desert and oasis in northwestern China, the nomadic Kazakh people living in cold highlands have been mainly engaged in agriculture and livestock (Han and Cho, 1997; Son, 2005). Traditional male and female clothing in these regions reflected the necessity for adapting to the natural environment and daily life; cotton and leather were mostly used as clothing materials for the purpose of keeping the body warm (Duan, 2004).

During the period of Song Dynasty in China (960–1279), Islam began to spread in earnest as Arab and Persian merchants migrated to Xinjiang Kazakh and established mosques. In the thirteenth century, due to Genghis Khan's expedition to the western regions, Muslims from Arab and Persian regions migrated to Kazakh and other Xinjiang regions along with Mongolian troops. They served in governmental offices or engaged in trade, and settled down with Islam and its culture taking root through marriage with Chinese Han women. Through this process, Islam spread throughout Kazakh around the sixteenth century (Xinjiang Uygur Autonomous Region Association for foreign cultural exchanges, 2012). However, Islam, which spread to Kazakh, continued to embrace traditional Chinese culture and formed its own religious culture, establishing unique characteristics of Islam in other countries such as the Arab (Park, 2006). The development of the Silk Road further strengthened the interactions between Kazakh and the Han nationality in the Central Plains. Kazakhs are deeply influenced by Chinese culture, especially the “Chinese cultural circle” with Confucianism at its core. The concept of “it is improper for men and women to touch each other's hand in passing objects” in Confucian rules of etiquette also clearly emphasizes the importance of women's chastity. It fully embodies the value of “serving for etiquette” in women's traditional clothing culture.

Eventually, elements of Islam and Confucian style were added to the Kazakh traditional clothing, resulting in their own distinctive style. The form, color, and decoration of Kazakh women's traditional clothing are the typical examples of the integration of oriental and western art.

## Influence of multiculturalism on Kazakh women's clothing

This study analyzed the form, colors, and decoration of clothing collected in 'Xinjiang Uygur Autonomous Region Museum, Aksai Kazakh National Museum, and Capital Museum based on a literature review and field study. Our research probed into the multicultural integration of Kazakh culture and cultures in regions along the Silk Road to interpret and decipher the clothing. Through these efforts, we are dedicated to promoting the inheritance and development of traditional and regional cultures along the Silk Road. Kazakh women's clothing features and the influence of the Central Plains culture and Arab culture were categorized and analyzed. We found that the form, color, and decoration of Kazakh women's garments, headdresses, and footwear show the fusion of foreign art styles.

In this study, 30 items each of women's garments, headdresses, and footwear types, totaling 90 items, were selected and their clothing image was analyzed from the perspective of form, color, and decoration. The classification criteria for dresses were based on pieces (whether one-piece or two pieces; Cho and Kim, 1992) and headdress type based on the shape and style (cylindrical, conical, draped, and mixed; Hong, 1986; Kim, 2001; Kang and Cho, 2015). Shoes were classified into normal types without shoe necks and boot types featuring raised shoe necks (Xu and Bae, 2015). Color classification of garments and accessories was examined and determined based on the colors occupying more than 50% of the entire area of clothing. If garments and accessories had a mix of several colors in a relevant ratio, they were classified as multiple-colored garments. The decorations were classified into no-patterned clothing and patterned clothing. The pattern decoration included embroidery and applique work.

### Multiculturalism in garment

From the perspective of cultural anthropology, different social customs and cultures accordingly breed different costume cultures and art. The *Book of Han* mentions that different terrain and natural environment create different lifestyles ranging from agriculture to animal husbandry (Ban, 2006). Therefore, the nomadic lifestyle of Kazakh ancestors in the plateau area determined their choice of attire, clothing materials and styles (Xu, 2016). According to literature, Chinese Kazakh women's clothing materials were mostly silk, fur, and cotton. The *Records of the Grand Historian* shows that Zhang Qian's expedition to the western regions stimulated trade and cultural exchanges between the Han in the Central Plains and Rouzhi in the Xiyu (Sima, 2006). Along with the culture, the weaving technique of the Han traveled into Xinjiang. Since then, silk has been used more frequently as a textile (Sima, 2006).

**Figure 1:**
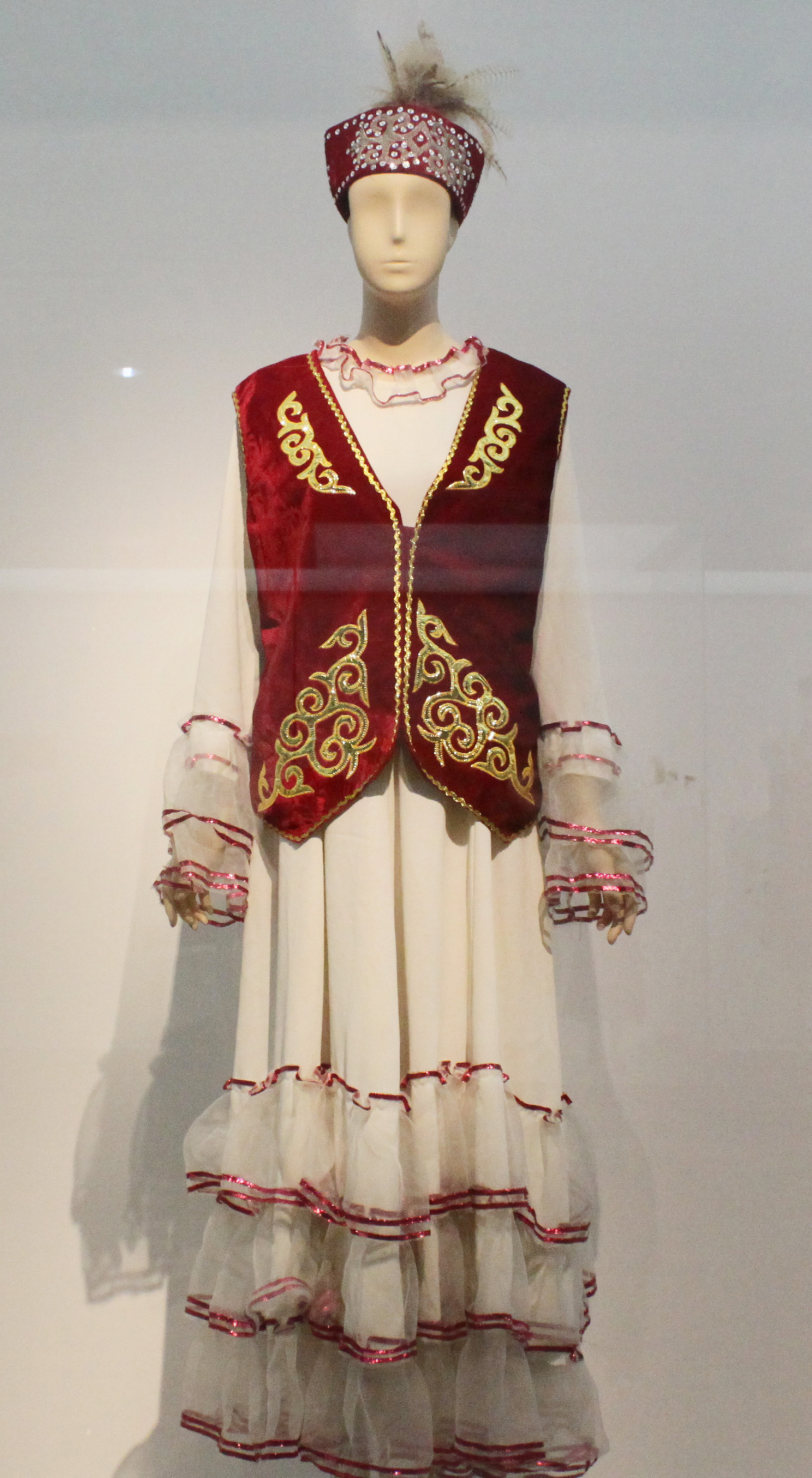
Garment in Kazakh. Figures 1, 2, 4, 5 are photoed by the author in Museum. Figures 1, 2 are from Capital Museum; Figure 3 is from Jeong (1997); Figure 4 is from Xinjiang Uighur Region Museum; Figure 5 is from Aksai Kazakh National Museum.

**Figure 2:**
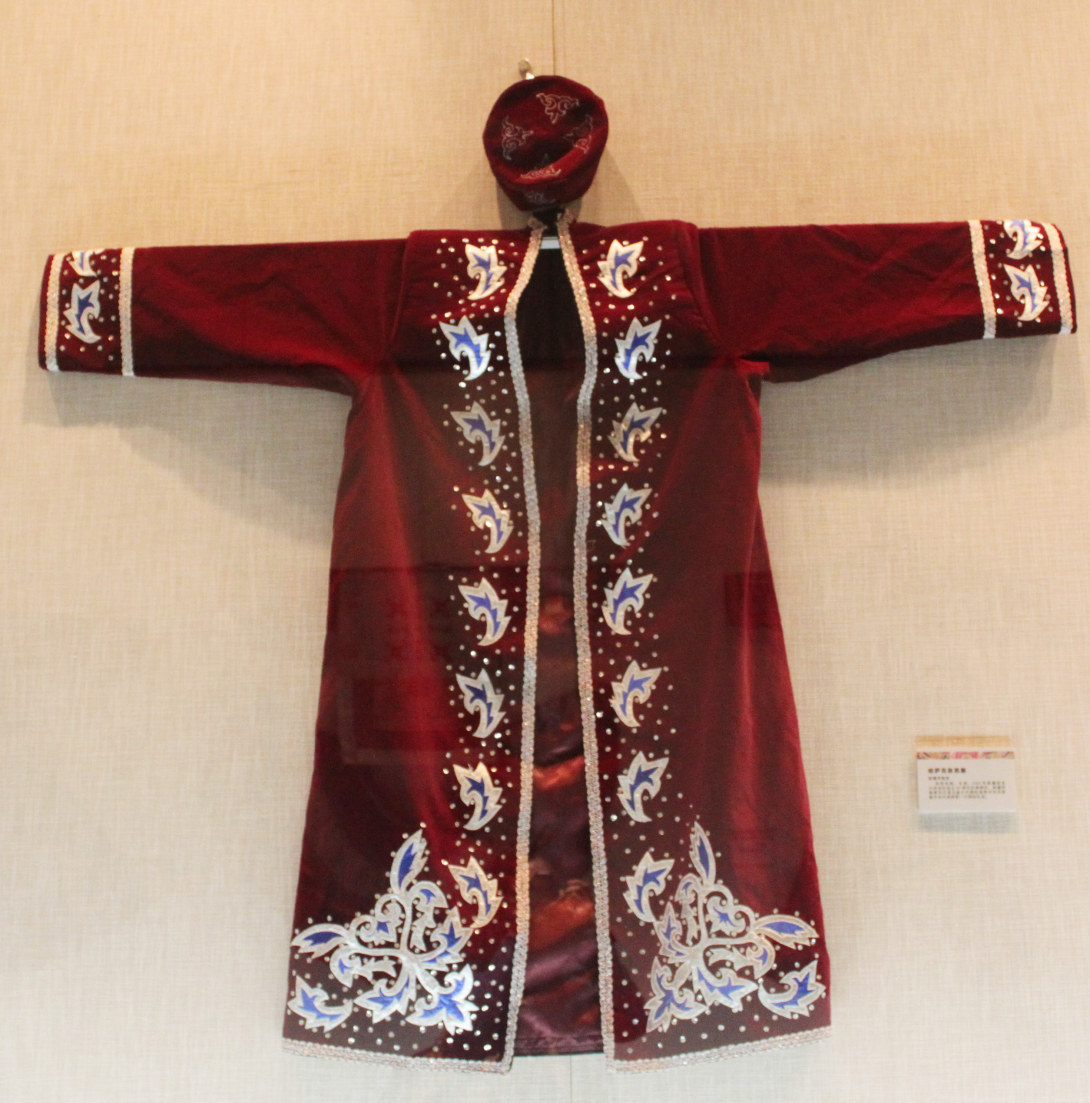
Waistcoat in Kazakh.

**Figure 3:**
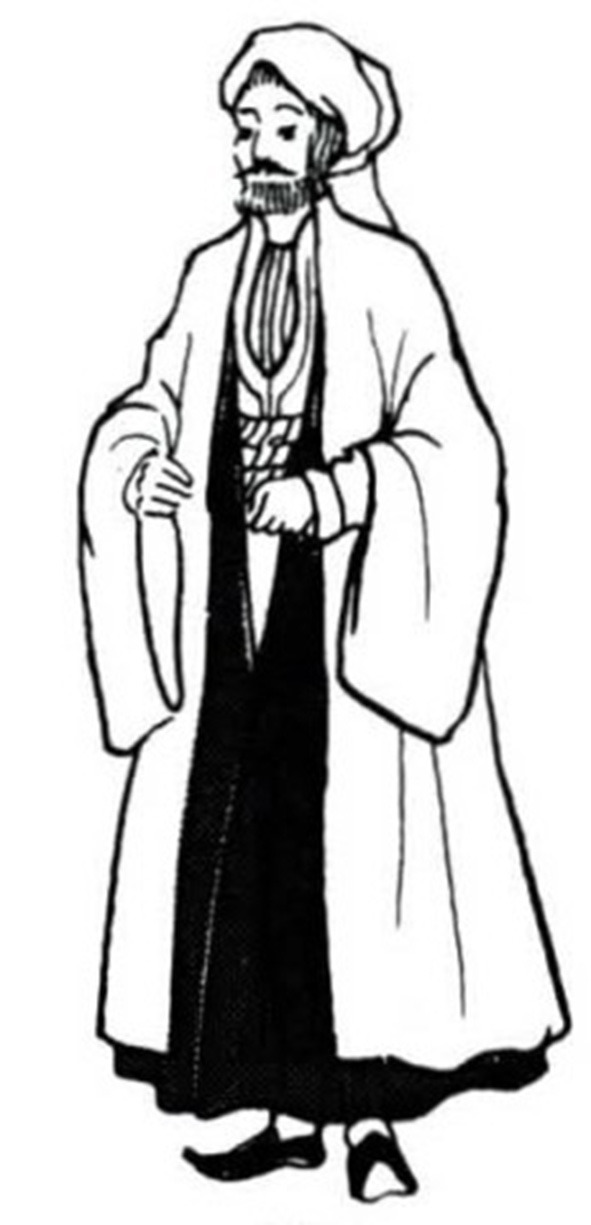
Jabbah.

**Figure 4:**
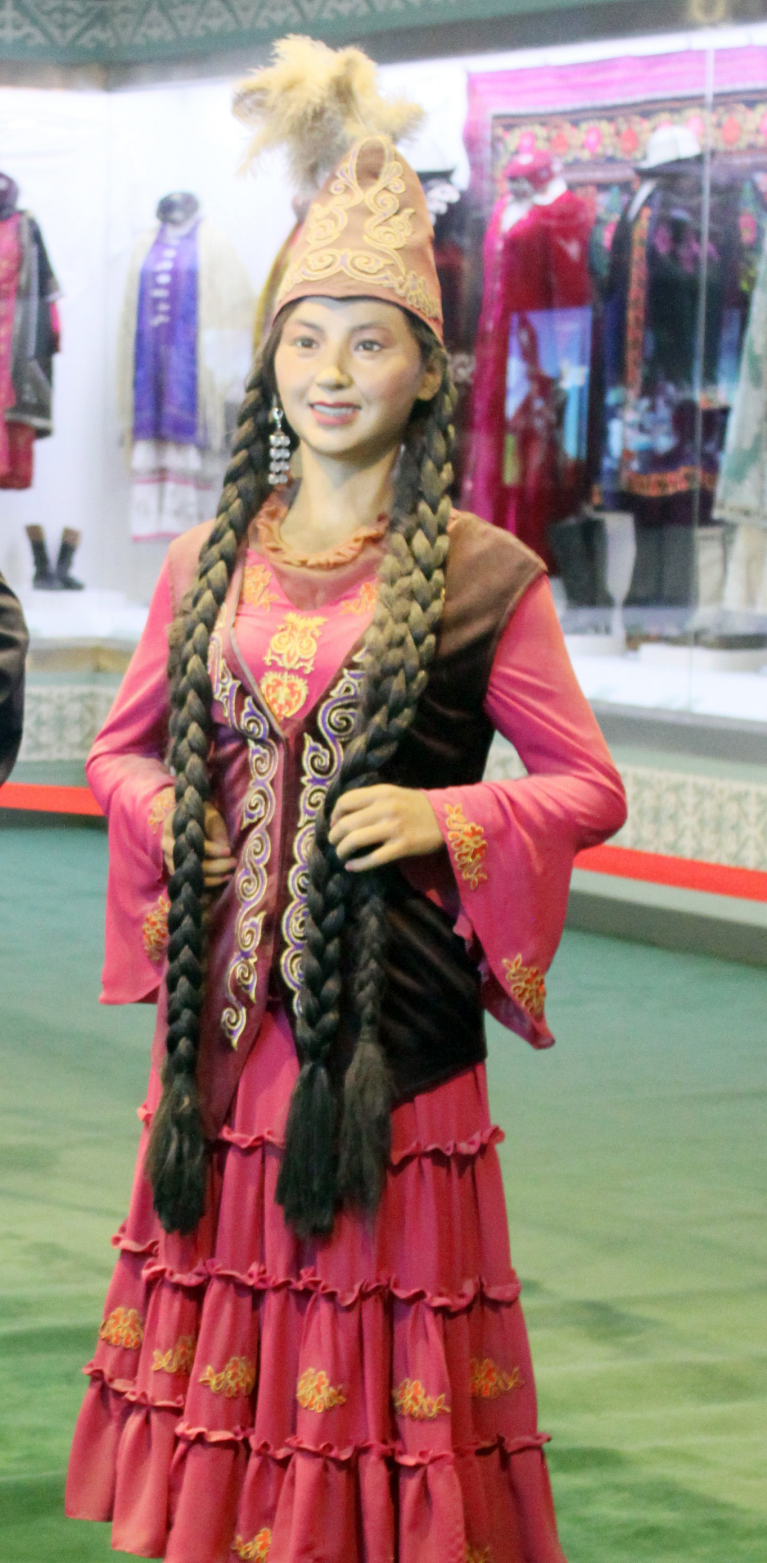
Garment in Kazakh.

**Figure 5:**
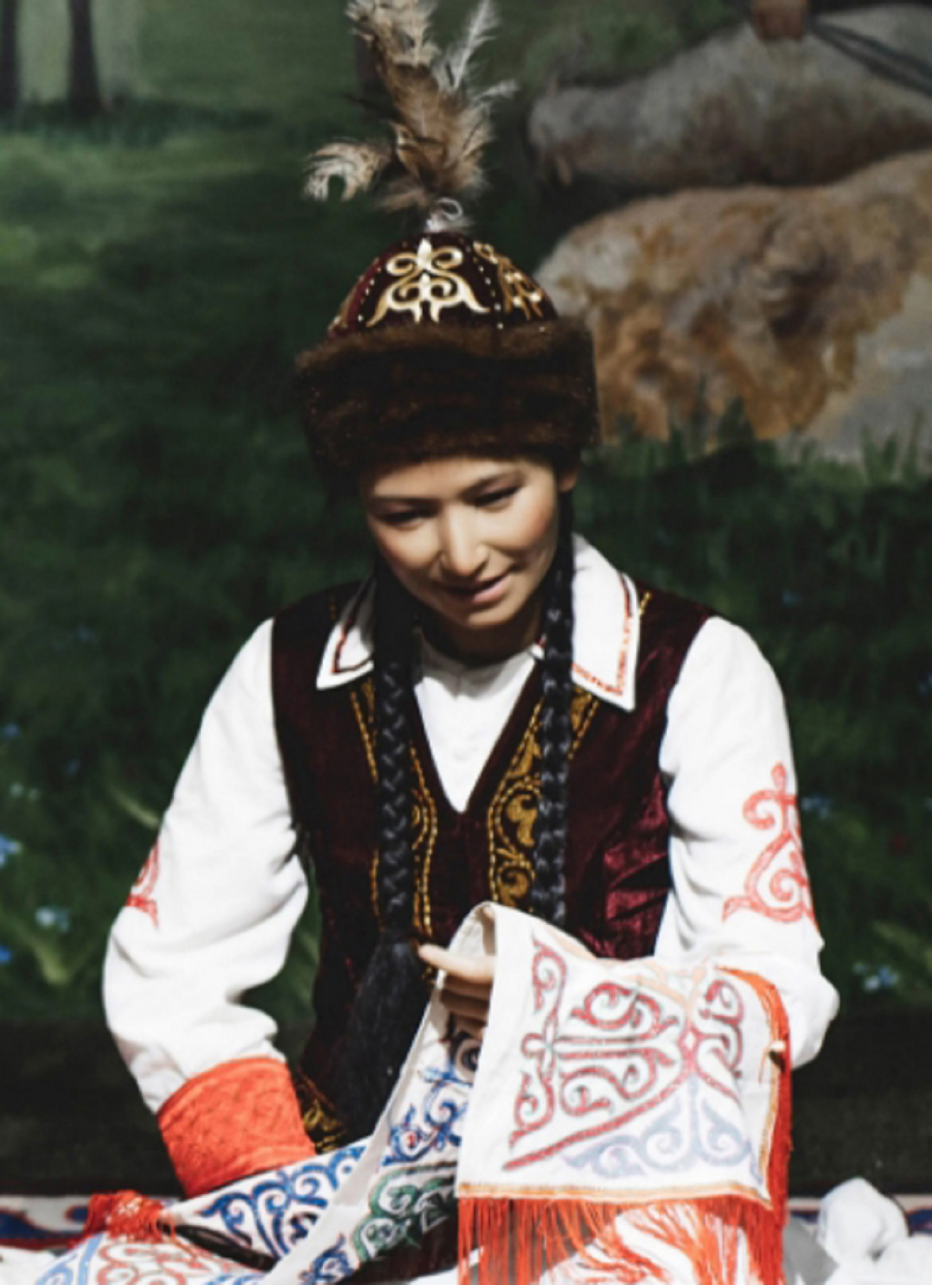
Swan totem embroidery.

All the 30 pieces of dresses were in the form of outerwear that was layered on a wide and long one-piece dress. Figure 1 shows the example of a Kazakh white silk one-piece dress and red leather waistcoat having a straight outline and open sleeve ends. The gowns were long enough to cover the feet. The waistcoat collar had a right-angle intersection, and the front of the clothing was embroidered with scroll patterns as seen in Figure 2. It can be seen that Jabbah in Islamic religious attire form, as in Figure 3, was highly relevant to the Kazakh's attires. Figure 4 shows a piece of Kazakh dress—a red cotton gown with a purple waistcoat, and the two pieces match each other as a set. The dress has a trumpet sleeve with a narrow shoulder but wide cuff, while the waistcoat edges are embroidered with owls, a traditional Chinese totem. In addition, the cuffs are often decorated with abstract white swan totem embroidery as in Figure 5. Dai Ping points out in the *Study of Chinese National Dress Culture*, that the Kazakh regarded the white swan as the symbol of their ancestors, representing that the totem for worshiping ancestors transforms into a visual symbol (Dai, 1994). To sum up, the characteristic square collar and round cuff of Kazakh women's dresses are the embodiment of the aesthetics of “Tian Yuan Di Fang” or the “Round sky and square earth” proposed by Confucianism and Taoism originating in the Han ethnicity. The scrolls pattern is adapted from traditional Chinese patterns. Japanese designer Kohei Sugiura describes in his book the *Birth of Modeling*: “scrolls pattern passes through ancient Greece to Rome and then to West Asia, India, and China in the same way” (Kohei, 1999). Thus, the Kazakh scroll pattern may have a similar origin to the arts of other cultures brought in along the Silk Road and gradually evolved into a unique style by absorbing the essence of many other cultures. Similar clothing and accessory designs have not been found in ancient Kazakh clothing. On the contrary, the design is pretty similar to ancient women's robes in Zhongyuan, Central China, and Zhongyuan clothing has integrated into the culture of ethnic minorities in the Northwest region (Xu, 2020). As seen in the archaeological image data of Han women's ancient robes, the collar is at right-angle and the sleeve cuff is wide and arc-shaped, and the scrolls pattern is embroidered on the dress by rolling the curving front robe along the waist, similar to the ancient robe with wide sleeves and winding fronts unearthed from the Tomb No. 1 of Mawangdui Han Tombs.

The colors used in the Kazakh women's garments were: red tint in 15 pieces (50.0%), white in five pieces (16.7%), blue tint in four pieces (13.3%), yellow tint in two pieces (6.7%), green tint in two pieces (6.7%), black in one piece (3.3%), and reddish violet in one piece (3.3%). The white color prominent in the Arab Islamic culture is particularly common in Kazakh married women's garments, which also reflects the Kazakh people's love for dairy products and living with livestock. Red, a popular color in Han ethnicity from Zhongyuan, and green, representing Islamic style, are widely used in the Kazakh unmarried women's dress (Zi, 2009). To be specific, red is the symbol of happiness and peace in Kazakh culture, which is a psychological state in traditional Chinese culture, while green embodies hope and the cultural connection with the Arab cultural influence.

Of the 30 pieces of garments, three did not have any decoration (10.0%) and 27 had different pattern decorations (90.0%) including embroidery and applique. Especially, patterns of lotus and scrolls are evident in their embroidery. Net embroidery are popular in the ornamental art of Kazakh women's clothing, as they are in their headdresses. It is worth noting that the lotus pattern in their dresses originated from Central Asia and entered into East Asian countries along the Silk Road by Genghis Khan in the thirteenth century. These are unlike the lotus pattern introduced into northwest China from India (Kim, 2004); the petals of Central Asian lotus are pointed and symmetrical with distinctive Arabic art styles. This ornamental art reflects the features of the times in Central Asia along the Silk Road.

### Multiculturalism in accessories

#### Headdress

The headdress is the most common accessory to protect the body. The *Notes of Lunheng* Department of History Peking University (1979) believes that the “Hat is more important than body clothing.” According to this record, the custom of wearing hats was born in ancient China and continues till today. Unlike men's headgear of various types, women usually cover their hair with a piece of headscarf or Jinzi. The *Shuowen Jiezi* (the ancient Chinese dictionary) explains that “Ze, the hair covered with the towel” (Xu, 2001), is a popular women's hairstyle in northwest China. According to *Tangshu* ·*Yufuzhi (The Book of Tang Dynasty*· *Chapter of Clothing)* (Liu, 1999), women's headdresses in northwestern China influenced the headdress in Tang Dynasty mixed with Central Asia, West Asia, and the Arab countries in the Silk Road, while the Kazakh women's headdress integrated the diversified artistic style, and has gone through several phases.

**Figure 6:**
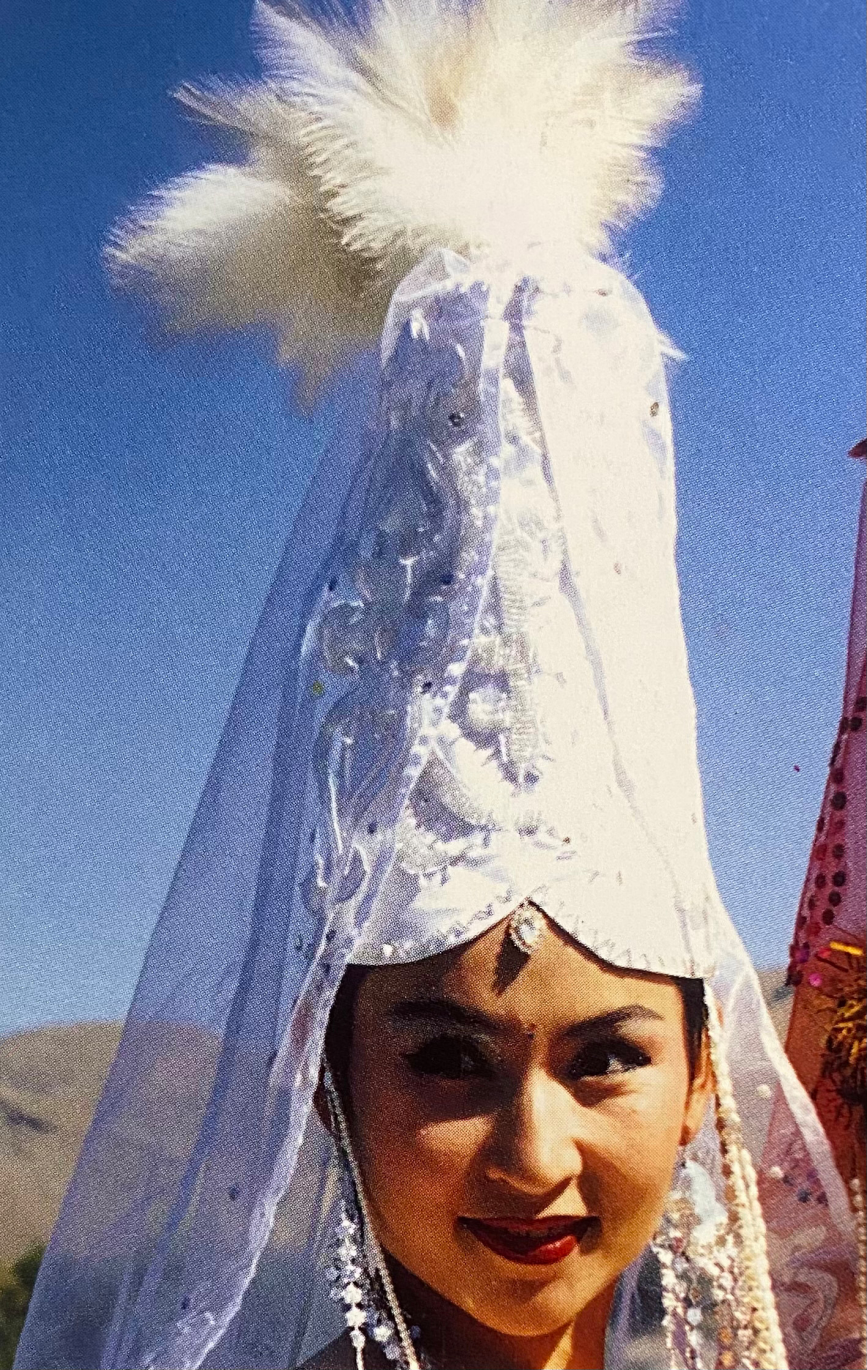
Combined-type Headdress of the Kazakh. Figure 6 is from Xinjiang Uygur Autonomous Region Association for foreign cultural exchanges; Figures 7, 10 are from Chang (2005); Figure 9 is from Xinjiang Uighur Region Museum; Figures 8, 11 are from Burqa (2013); Drapery-Type Headdress in Arab (2019).

**Figure 7:**
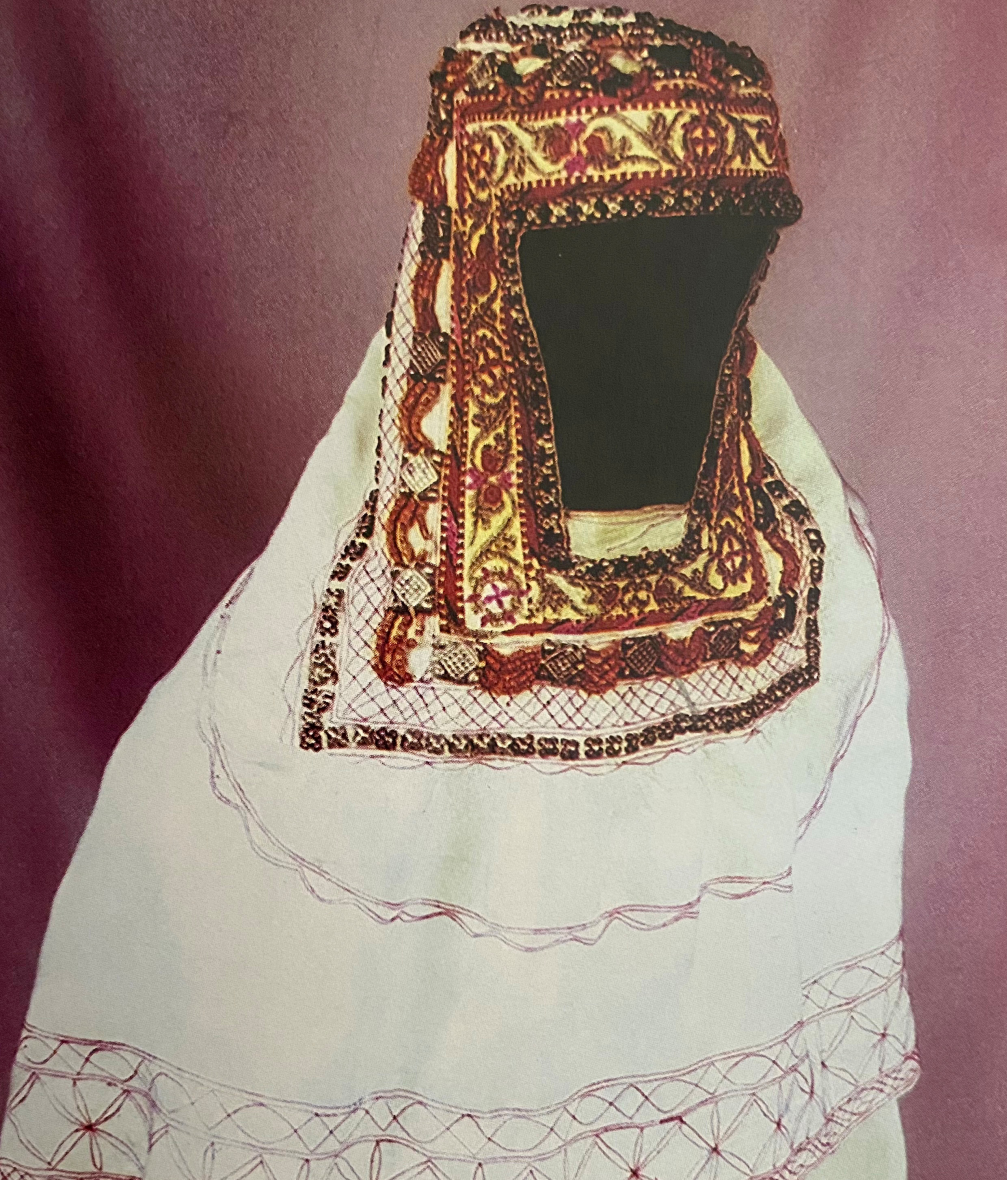
Combined-type Headdress of the Kazakh.

**Figure 8:**
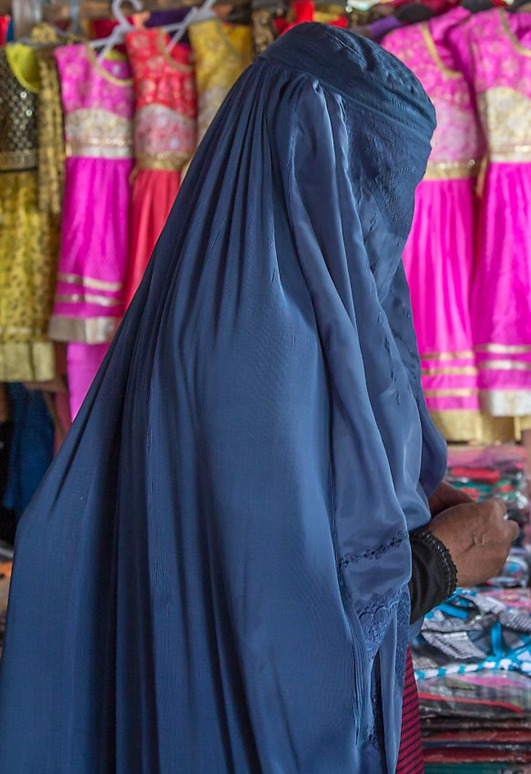
Burqa in Afghanistan and Iran.

**Figure 9:**
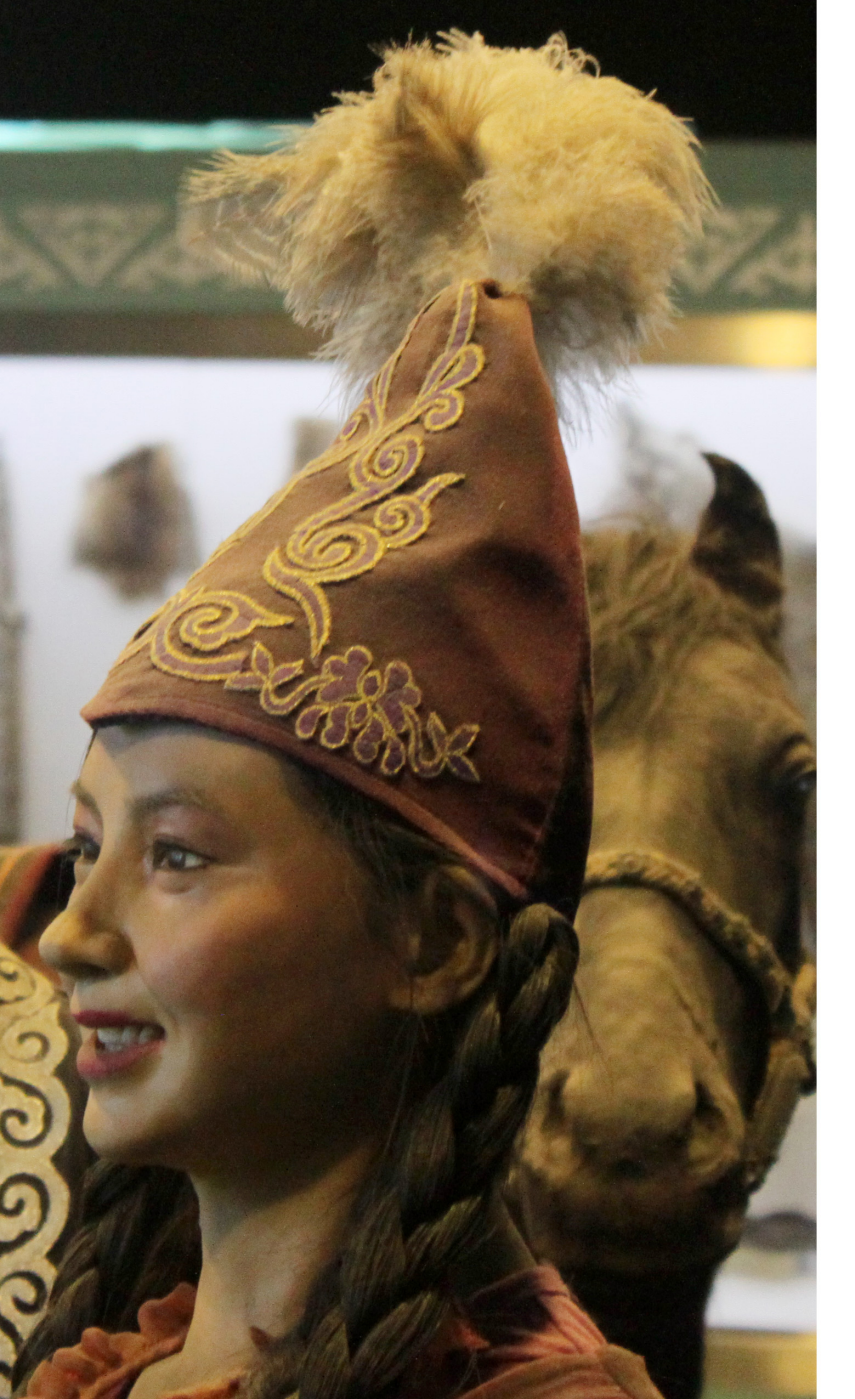
Conical-type Headdress of the Kazakh.

**Figure 10:**
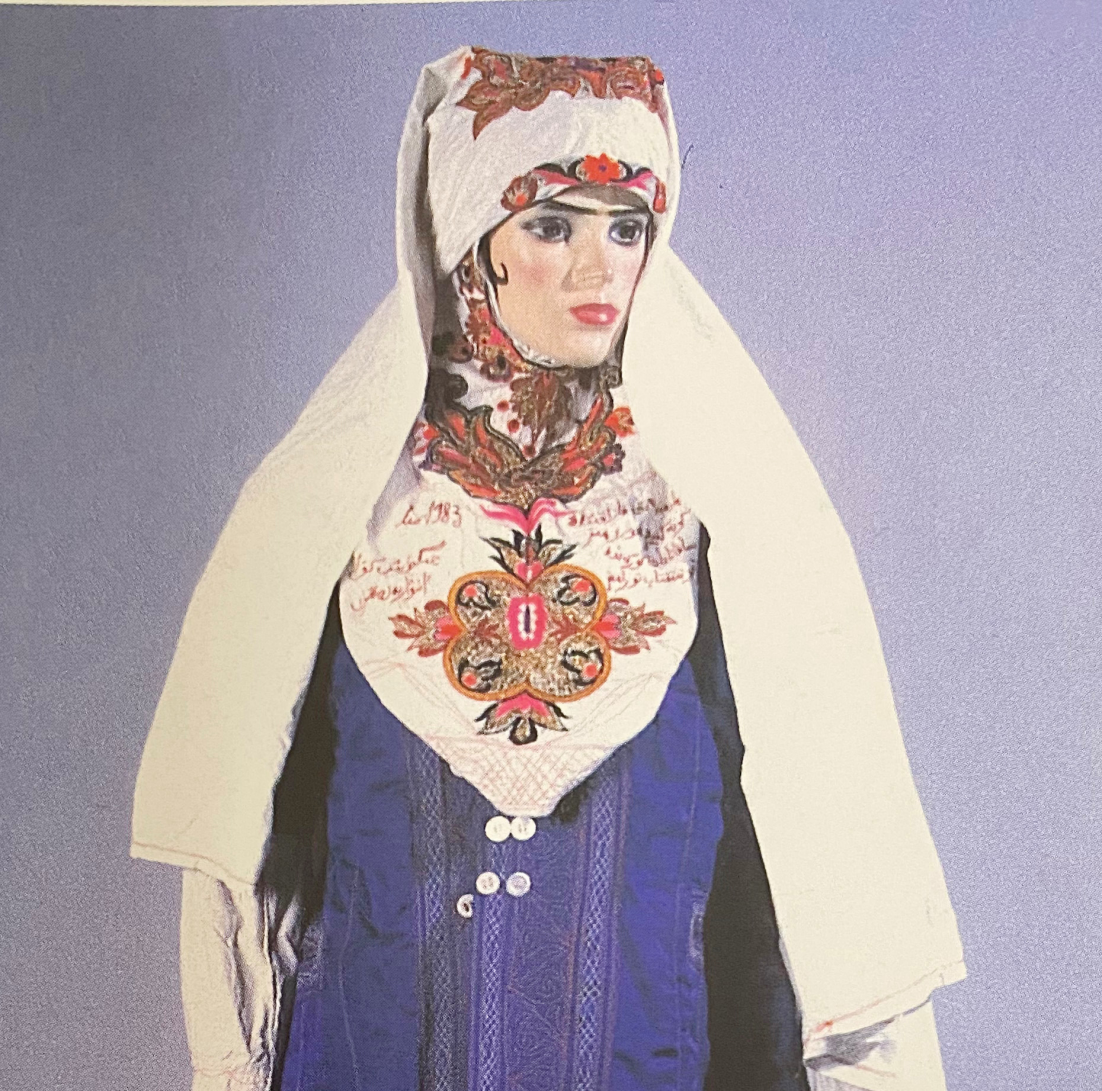
Drapery-type headdress in Kazakh.

**Figure 11:**
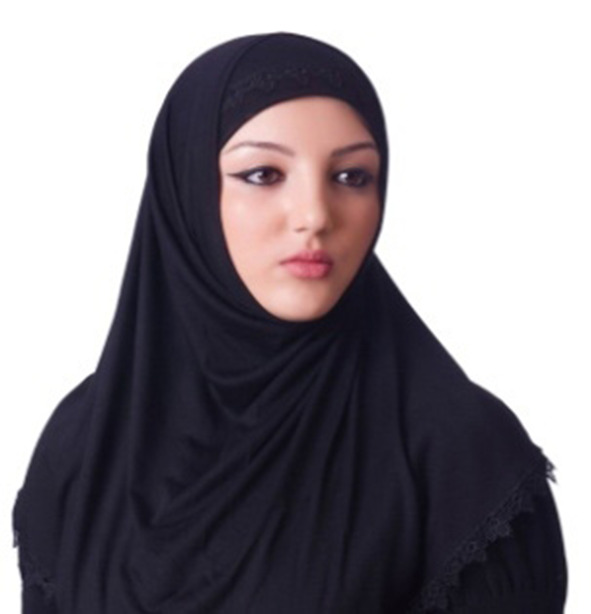
Drapery-type headdress in Arab.

Among the Kazakh women's headdress we examined, we found the following types: 12 pieces of mixed type (40.0%), eight pieces of cylindrical type (26.6%), five pieces of drapery type (16.7%), and five pieces of cone type (16.7%). In seven of the mixed-type headdresses, a combination of conical and drapery types was seen (25.9%); they had a long red veil suspended on a white cone-type headdress as shown in Figure 6. Mixed-type headdresses combining the cylindrical and drapery types were found in four pieces (14.8%), which had a cylinder-type headdress connected to a closed, drapery-type headdress covering the head, ears, chin, neck, and shoulders except for the face. The headdress seen in Figure 7 is an example of this type of combined-type headdress and embroidered with stripes and grid patterns on a white background. The design of this headdress is similar to that of a burqa (Figure 8), but differing only in the absence of a veil covering the face, and the length of the burqa that covers the women's entire body from head to toe. The headdress seen in Figure 9 is a conical-type headdress made of red-colored animal skin and is decorated with feather decorations and owl patterned applique. Behind the design of the high-top hat is the primitive belief that “people can communicate with God only by standing high” in Shaman folk culture (Kim, 1998). The drapery-style headgear in Kazakh has two ends of the drape pulled back behind the head while the outer layer overlaps and fixes the headdress with the edges falling naturally as in Figure 10. The style is similar to the women's drapery-type headdress in Arabic culture covering the hair, including on the forehead, ears, neck, and shoulders as in Figure 11. It can also be noticed that the drapery-type headdress seen in Figure 11 is irregular in form without being sewn, compared to the more elaborate Kazakh headdress seen in Figure 10.

The colors used in the headdresses were: white in 13 pieces (43.3%), red tint in 10 pieces (33.3%), blue tint in four pieces (13.3%), and multicolored in three pieces (10.0%). Most of the headdresses of married women are white, and the headdresses of unmarried women are red. As for ornamental patterns, all 30 pieces had animal and plant patterns and were decorated including applique and embroidery. In particular, Kazakh women's headdresses often depicted patterns originating from people's daily walks of life, such as antlers, goat horns, ox horns, or other animals, or scrolls embroidered in Arabic style. The animal patterns were mostly figurative horns, reflecting the philosophical view of “harmony between man and nature” in Taoism, the foundation of Chinese civilization. As a manifestation of the primitive and traditional religious beliefs, the pattern and the headgear show the assimilation of traditional folk style as advocated in the “Chinese Cultural Circle.” In conclusion, Kazakh women's headgear was influenced by foreign cultural elements due to the communication and cultural exchanges brought by the Silk Road, and while integrating the diversified styles that originated in different cultures, it also retained the heterogeneous factors of the Han traditional culture.

#### Footwear

In addition to supporting the human body, the foot contains unique cultural connotations, such as being trapped, pure, strong, and other metaphorical meanings (Chen, 2013). Therefore, just like clothing, the design of the footwear is symbolic and its importance is self-evident. From the perspective of the integration of foreign culture introduced through the Silk Road and Chinese traditional art, the footwear in ethnic minorities in Northwest China is mostly high-cut boot shoes, particularly the shape and decoration of Kazakh women's knee boots. According to historical records, the boots met the nomadic life demands and have been used in the ancient western region. As stated in the *Chinese Commentaries on Antiquity and Today*: “Boots are from the West Hu in ancient times (Ma, 1989).”

**Figure 12:**
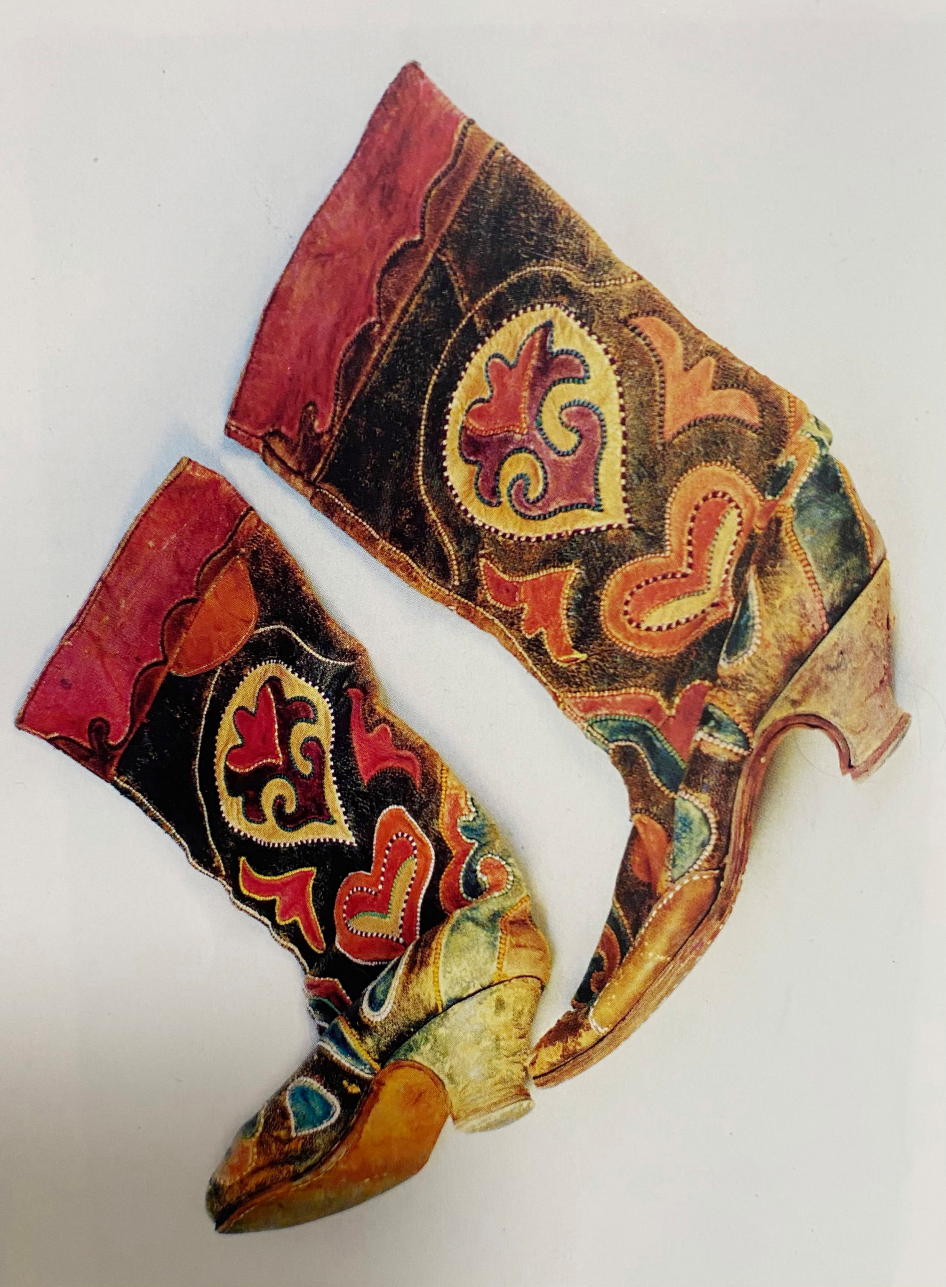
Boots in Kazakh. Figure 12 is from Xu (2015); Figure 13 is from National Museum of Korea (2018).

**Figure 13:**
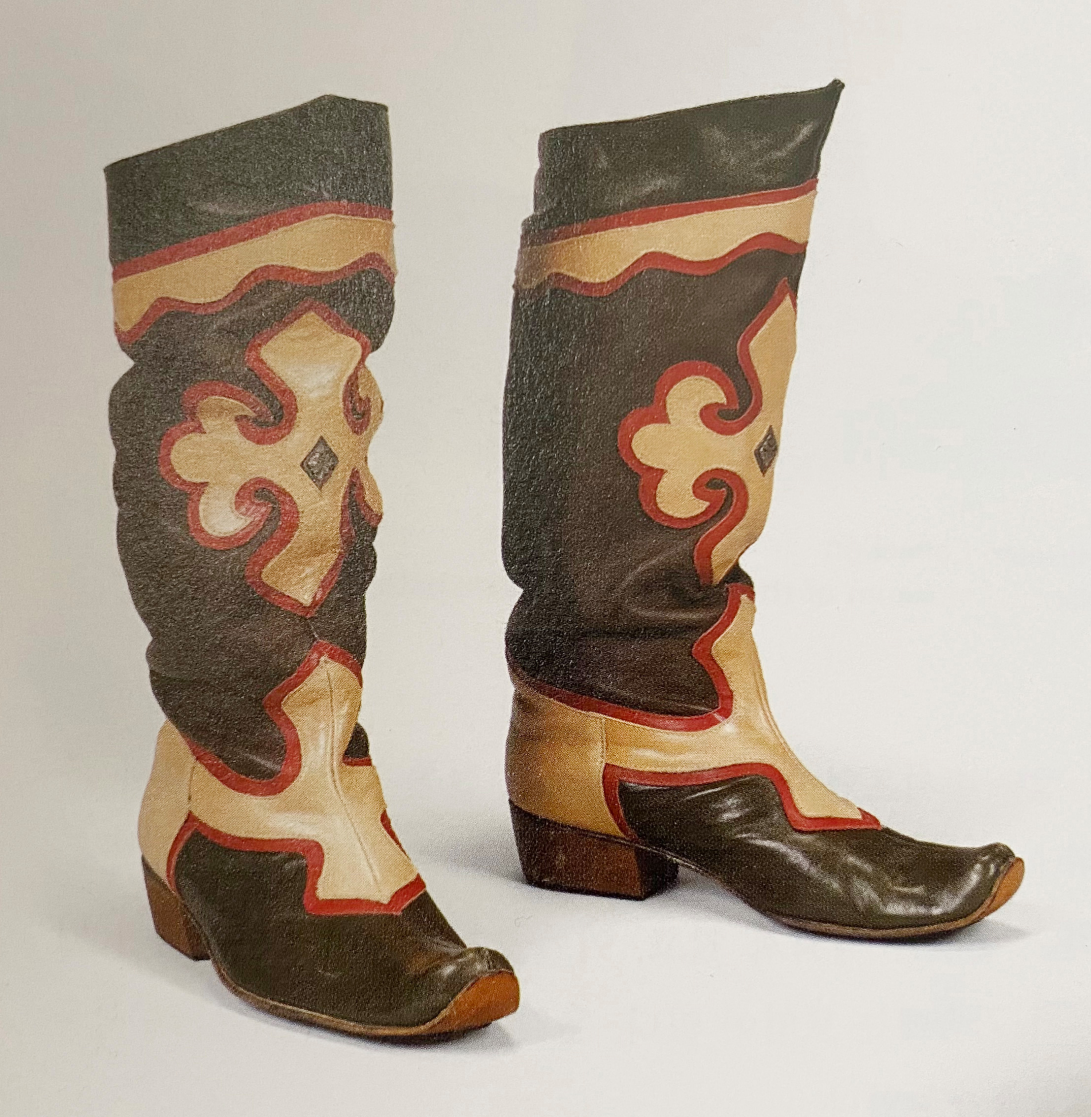
Boots in Kazakhstan.

All 30 Kazakh women's footwear was found to be boots, which seems practical given the need to keep the feet warm from the cold due to the cold climatic conditions in the region. The upturned shoe mouth of women's boots in Kazakh is very common, as in Figure 12. The shape of the upturned shoe mouth can be regarded as a manifestation of the ethics and morality principle “walk the right way with integrity in heart” at the core of Chinese civilization. The shape of the shoe's mouth, heel, and upper edges are similar to the women's boots in Kazakhstan, Uzbekistan, and other Central Asian regions as seen in Figure 13. Clearly, the women's boots in Kazakh were more or less influenced by the shoe culture in Central Asian cultures along the Silk Road (National Museum of Korea, 2019). However, this kind of upturned shoe head is also inherited from the curved top angle shape of the ancient Chinese buildings and echoes the “Looking up to observe the universe” written in the *Book of Changes* (Sun, 2021), highlighting Chinese traditional culture.

In terms of color, black and brown tints were very common in women's boots. The colors used in the Kazakh women's boots were: black in 17 pieces (56.7%), brown tint in six pieces (20.0%), red tint in six pieces (16.7%), and white and blue tints one piece each (1.1%). Black with strong Arab Islamic style symbolizes dignity and silence (Lee, 1986), indicating Kazakh belief.

As for ornamental patterns, all 30 pieces of women's boots had animal, plant, and geometrics pattern decorations, using applique work. The boots' upper portion was decorated with an eagle totem as in Figure 12, a “unique symbol in the ethnicity.” The Encyclopedia of China · Religion recorded that “It is said that the first Shaman of the Siberian Buryat was a talking eagle whom the gods of heaven sent to protect the clansman...... (thus the eagle) is the original Shaman (Editorial Board of China Encyclopedia, 2002).” It is enough to prove the status and significance of eagle totem in Kazakh people's daily life. Such eagle patterns are often used with the lotus patterns introduced from Central Asia to Northwest China, showing similarities shared with ornamental clothing art discussed above. In conclusion, apart from the culture brought by the Silk Road, Kazakh women's boots also display Kazakh traditional culture and primitive beliefs through ethnic art. As a result, Kazakh women's footwear pattern fully shows the ethnic features of the western region, including elements of foreign cultures, but behind it is the close connection to national spirits.

## The value of Kazakh women's social role from the perspective of diverse clothing culture

From a multicultural perspective, Kazakh women's clothing embodied the core content of the social role and value of that time, be it gender socialization, social initiatives and other characteristics that emphasize the value of women's social role. It represented the cultural space and fashion of its society such that the value of women's social role in that era could be understood by the shape, color, and pattern of clothing they wore then.

### Clothing form: Social restriction and national identity recognition

Kazakh women's clothes had coats that matched with their loose and long dresses. This is persumed to be highly relevant to the religious practices of Islam, which expects the body to be covered with closed forms of garments. In Kazakh women clothes, this takes the form of a long coat that is wide and long enough to fall below the knee, a design that remarkably close to the formative feature of the Islamic Jabbah.

The types of women's headdresses were overall the mixed type with 12 pieces (40.0%). Further, eight pieces were cylindrical type (26.6%), five pieces were drapery type (16.7%), and five pieces were conical type (16.7%). Since a drape is included in the mixed type of headdresses, it can be said that the percentage of headdresses with drapery was 56.7%. Therefore, it can be seen that their specific forms are highly relevant to the closed form covering the head, which is a characteristic of Islamic attire. Conservative clothes like garments and headdresses are designed to show the most charming side only to their husbands, which has many similarities with the traditional Chinese Confucianism that “it is improper for men and women to touch each other's hand in passing objects.” This was a positive social demand and has the social function of maintaining moral order. In addition, the headdress worn by Kazakh women is no longer the identification of marriage or religion, but a prominent external dress image that conforms to the Islamic Koran, highlighting its differences, while still enhancing the sense of national identity. Besides, in terms of social practice, women are not completely passive followers of tradition, but leaders in maintaining tradition. With the strong support of the state and the government, more Kazakh women are involved in public service and family affairs decision-making. Women's roles are changing, their status is improving, and their sense of equality is becoming stronger. Therefore, by combing and understanding the group culture of “women” in Kazakh religion, culture, nationality, and other aspects, we can find a direction for the research on “women's role and status” in Kazakh clothing.

### Color and pattern of clothing: The improvement of self-consciousness and the remodeling of women's social gender roles

The color and pattern of clothing, as the cultural marks that distinguish genders, endow special social gender meaning and strengthen the consistency between physiological gender and social gender (Wang and Liu, 2011). According to the analysis and investigation of chromatics, 35% of people believe that blue can best represent men, only 16% think that the color representing women is red, and <13% think that it is white (Aiwa, 2004). Therefore, it can be said that red and white are not the best colors representing women in people's traditional mindset. As mentioned earlier, the colors used in the Kazakh women's garment and headdress are 50.0 and 33.3% of red tint, which symbolize enthusiasm, and 16.7 and 43.3% of white, which symbolizes purity. Blue, the symbol of wisdom in Islam, appeared in 13.3%. The result of such clothing color selection not only reflects that Kazakh women are influenced by Islamic and Confucian cultures, but also transcends gender differences and breaks away from men's values and aesthetic system, which is a new trend of the development in gender relations in modern society and an improvement of women's self-awareness.

In addition, embroidery and applique are mainly used for the decoration of women's garment, headdress and shoes. Such embroidery and appliquer techniques have gradually become the main breadwinners of Kazakh families. In this process, women began to pay attention to their social value, and they are no longer limited to their family life, but have also gradually changed the traditional concepts of women's role, ability and value, and guided the social role of women from traditional Kazakh women to diversified roles in society. Therefore, the evolution of this kind of women's value is also reflected in Kazakh women's life. Although the process is extremely slow and weak, the intensity of the transformation in women's clothing perception is relatively significant and forms the value of social progress. Therefore, it is meaningful for the whole Kazakh community which in turn is conducive to the promotion of women's self-awareness and social value to the greatest extent.

## Conclusion

The Kazakh women's traditional dress culture integrates the garment and accessory characteristics of cultures along the Silk Road, showing cross-regional and cross-cultural influences. As clothing is the embodiment of “culture, history, and society,” it is the best envoy between Silk Road cultures. This paper mainly explored the collision of foreign cultures and researched the alien culture fusion against the background of the “silk road.” We also analyzed the Kazak women's dress brought about by the “free women” grit and other social and historical values to showcase the historical and cultural heritage and revitalize its development.

This study aimed to find out the characteristics of Kazakh women's dress image in China from a multicultural perspective, so as to clarify the position and social structure of Kazakh women in social life. First, from the perspective of clothing form, the Kazakh women's garment was multi-layered. The most representative headdress of Kazakh women was a drapery-type headdress wrapping the head, so it can be identified that their clothing and headdress were very relevant to the formative closing characteristic of Islamic religious attire. Such a garment form can effectively maintain moral order and improve women's sense of national identity. Secondly, from the perspective of clothing color, it was found that the Kazakh women's garment and accessories often used not only black, white, and blue tints, which were symbolic colors in Islamic religious attire but also red tints, which were favored by the Chinese people. The choice of such colors also proved that it transcended gender differences and broke away from men's values and aesthetic system. Finally, from the perspective of clothing patterns, embroidery, and pasting techniques were mostly used, which provided women more opportunities to participate in social events and space for women's development. Such status promotion can awaken women's independent personalities and shape their image in the new era.

An in-depth multicultural analysis of the characteristics of Kazakh women's clothing will help us understand the background of clothing culture formation from a female social perspective. However, this article only focuses on the theoretical basis of the overall view of culture and examines the cultural interoperability between its clothing art form and society, which may not be comprehensive. We hope that this study contributed to the understanding and preservation of the clothing culture of Chinese minorities which is disappearing. By examining the association between the clothing of other Chinese minorities and the religious clothing other than the Kazakh women's clothing, this study has hopefully showcased the intersection of eastern and western cultures in history, and thrown some light on the relationship between religion and clothing that has existed for thousands of years.

## Data availability statement

The original contributions presented in the study are included in the article/supplementary material, further inquiries can be directed to the corresponding author/s.

## Author contributions

RX: manuscript draft and revision.

## Funding

This study was funded by the General Research Project on Philosophy and Social Sciences in Jiangsu University *A Comparative Study of Chinese Kazakh Clothing on the Silk Road and Central Asian Kazakh Clothing*, Project No. 2020SJA0533; High-level Scientific Research Initiation Project of Jinling Institute of Technology *Study on the Motif of Ethnic Minority Dress Decoration in Northwest China under the Background of One Belt and One Road*, Project No. jit-b-201920; and National first-class disciplines program funding.

## Conflict of interest

The author declares that the research was conducted in the absence of any commercial or financial relationships that could be construed as a potential conflict of interest.

## Publisher's note

All claims expressed in this article are solely those of the authors and do not necessarily represent those of their affiliated organizations, or those of the publisher, the editors and the reviewers. Any product that may be evaluated in this article, or claim that may be made by its manufacturer, is not guaranteed or endorsed by the publisher.
